# Effects of short-term exposure to air pollution on respiratory symptoms and lung function among patients with asthma

**DOI:** 10.4103/mgr.MEDGASRES-D-25-00164

**Published:** 2026-03-06

**Authors:** Yixuan Liao, Qin Cheng, Steven Kenneth Huang, Xuan Yang, Xuan Yang, Wenjun Mi, Wanlu Sun, Minxia Li, Guohua Jia, Wanzhen Yao, Furong Deng, Yahong Chen

**Affiliations:** 1 Department of Pulmonary and Critical Care Medicine, Peking University Third Hospital, Beijing, China; 2 Department of Pulmonary and Critical Care Medicine, Beijing Hospital, National Center of Gerontology, Institute of Geriatric Medicine, Chinese Academy of Medical Sciences, Beijing, China; 3 Department of Pulmonary and Critical Care Medicine, University of Michigan, Ann Arbor, MI, USA; 4 Department of Occupational and Environmental Health Sciences, School of Public Health, Peking University, Beijing, China; 5 National Center for Chronic and Noncommunicable Diseases Control and Prevention, Chinese Center for Disease Control and Prevention, Beijing, China

**Keywords:** adult, air pollution, Asthma Control Test, asthma, forced expiratory flow, forced expiratory volume in 1 second, lung function, mixed effects model, respiratory symptom, short-term exposure

## Abstract

Since air pollution can cause acute exacerbation of asthma, we aimed to investigate the effects of short-term exposure to ambient air pollution on respiratory symptoms and lung function among patients with asthma. A prospective and observational panel study recruited 32 patients with asthma from November 2015 to December 2016. The Asthma Control Test scores and lung function measurements of the patients were repeatedly assessed. Daily ambient air pollution data, including particulate matter (PM_2.5_ and PM_10_), sulfur dioxide, nitrogen dioxide, and carbon monoxide, were obtained from nearby central air-monitoring stations. Mixed effects models were used to examine the associations between pollutant exposure and health measurements. Clinical data were repeatedly collected 2 to 13 times each month, totaling 266 visits. Increases in the interquartile ranges of PM_2.5_ (74.5 μg/m^3^, 6-day), PM_10_ (78 μg/m^3^, 5-day), sulfur dioxide (10 μg/m^3^, 7-day), nitrogen dioxide (37 μg/m^3^, 6-day), and carbon monoxide (0.7 mg/m^3^, 6-day) levels were significantly associated with reductions in Asthma Control Test scores by 3.6%, 2.8%, 3.2%, 3.8%, and 2.9%, respectively. An interquartile range increase of 78 μg/m^3^ in the 7-day moving average concentration of PM_10_ was significantly associated with reductions of 3.6% in percentage of predicted forced expiratory volume in 1 second and 6.0% in percentage of predicted forced expiratory flow at 25% of the forced vital capacity, respectively. Short-term ambient air pollution may aggravate respiratory symptoms and cause a decline in lung function among patients with asthma.

## Graphical abstract

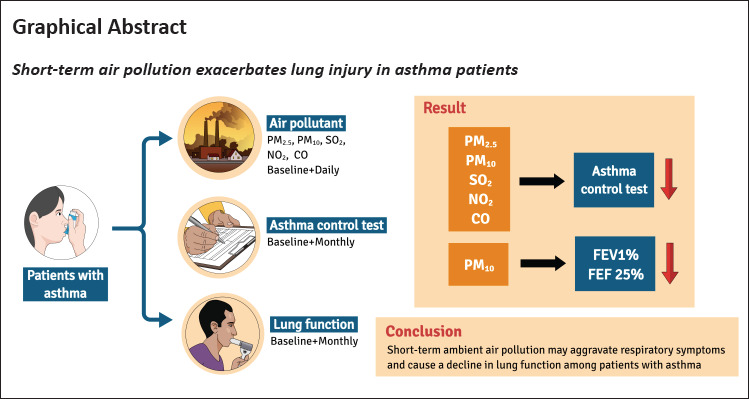

## Introduction

Asthma is a common chronic respiratory disease affecting all age groups worldwide. It is a heterogeneous disease commonly characterized by chronic airway inflammation. The respiratory symptoms of asthma, such as wheezing, shortness of breath, chest tightness, and cough, usually vary in frequency and intensity over time, and airflow limitation is variable and reversible.^1^ According to the global burden of disease study, there were 41,555,628 incident cases of asthma worldwide in 2021, posing a heavy disease burden.^2^ Air pollution is an important risk factor in asthma pathogenesis, comprising both natural and anthropogenic pollutants. Sulfur dioxide (SO_2_), nitrogen oxides (NOx), carbon monoxide (CO), and particulate matter (PM) from fuel combustion or motor vehicle emissions are the most common outdoor air pollutants.^3^ Previous studies have demonstrated that exposure to air pollutants has harmful effects in both children and adults. Multiple environmental exposures together increase incident asthma risk throughout the life.^4^,^5^ Increased air pollution leads to a rise in medication use and an increased frequency of exacerbations, emergency visits, and hospitalization of patients with asthma.^6^,^7^,^8^,^9^ Although these associations have been well-established,^10^ the role of air pollution in asthma development remains unclear.

Asthma is characterized by variable symptoms, which adversely affect quality of life and contribute to disease burden.^11^,^12^,^13^ Poor symptom control is significantly associated with an increased risk of asthma exacerbations. The Asthma Control Test (ACT) is a standardized, self-administered assessment tool that provides a practical measure of overall asthma control.^14^ It is simple and can be easily completed by patients,^15^ enabling physicians to easily evaluate the asthma control level. The ACT comprises four questions related to symptoms and reliever use, as well as one question on the patient’s perceived asthma control.^16^ Previous studies have used the ACT score as an asthma health outcome measure for asthma in relation to air pollution. He’s study^17^ found that day-to-day increases in personal PM_2.5_ and ozone exposure were associated with decreased individual symptom scores and total childhood ACT scores among children with asthma. Improvements in total childhood ACT scores were observed after interventions targeting desert dust and anthropogenic air pollution exposure after 3-month follow-up.^18^ Most prior research on the relationship between ACT scores and air pollution in asthma has focused on children. Maestrelli’s study^19^ was done in adults with asthma, but found only a weak association between increased 24 hours PM_10_ personal exposure and decreased ACT scores (*P* = 0.06).

Lung function is an important predictor of long-term asthma outcomes. Patients with asthma may experience an accelerated decline in lung function and progressive airflow limitation that may not be completely reversible.^1^ In children with asthma, air pollution adversely influences lung function and methacholine responsiveness.^20^,^21^,^22^ Nitrogen dioxide (NO_2_) and SO_2_ were constantly associated with decreased levels of peak expiratory flow in asthmatic children.^23^ Furthermore, most of these studies were conducted in countries with relatively low levels of air pollution levels and focused on children, resulting in limited prior research on the association between air pollution and lung function in adults with asthma living in cities with high air pollution levels.

Thus, a panel study was conducted to assess the associations between short-term exposure to high ambient air pollution and both respiratory symptoms, measured by ACT, and lung function in adults with asthma.

## Methods

### Study design and participant recruitment

**Figure 1:**
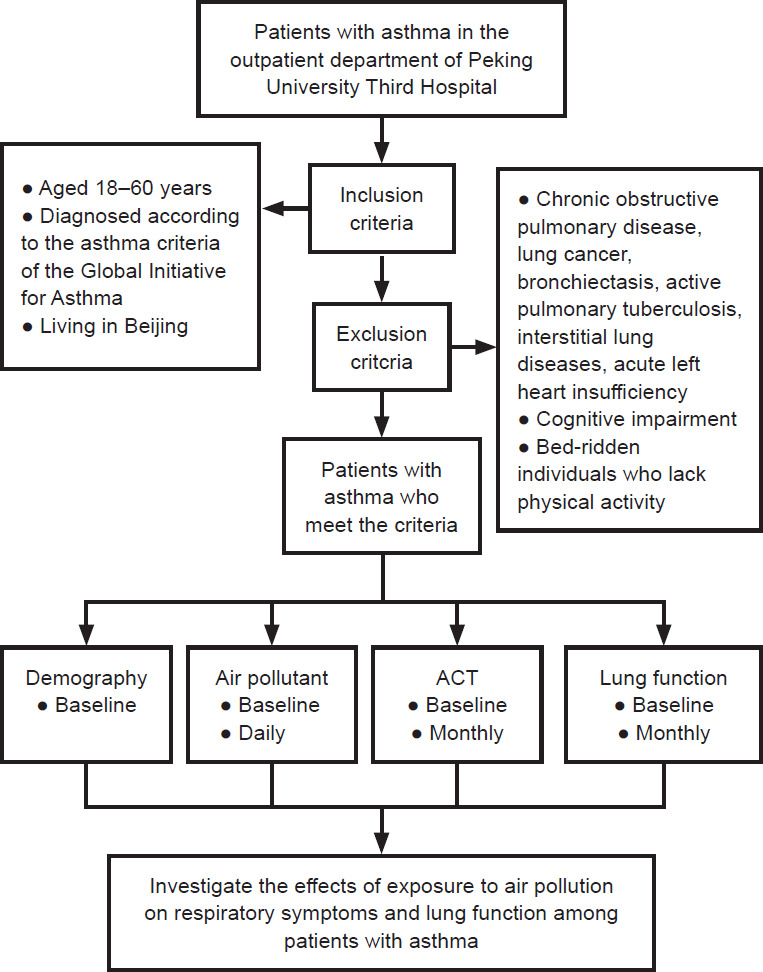
Study design flowchart. ACT: Asthma Control Test.

A prospective and observational panel study was conducted to evaluate the potential effects of short-term exposure to air pollution on health outcomes in patients with asthma. The study design flowchart is presented in **Figure 1**. The inclusion criteria were as follows: (1) age 18–60 years; (2) a diagnosis of asthma based on the Global Initiative for Asthma criteria^1^ (patients with wheezing, shortness of breath, chest tightness, cough, and lung function meeting at least one of the following: a positive bronchial reversibility test, indicated by an increase in forced expiratory volume in 1 second (FEV1) > 12% and > 200 mL from baseline after inhalation of 200 µg albuterol; or a positive bronchial challenge test, defined as a ≥ 20% decrease in FEV1 from baseline after methacholine inhalation at concentrations < 4 mg/mL); and (3) residence in the district of Beijing. Exclusion criteria included a diagnosis of chronic obstructive pulmonary disease, lung cancer, bronchiectasis, active pulmonary tuberculosis, interstitial lung disease, acute left heart dysfunction, cognitive impairment, or being bedridden with limited physical activity. Demographic data, including name, age, sex, address, smoking history, comorbidities, respiratory symptoms, and asthma symptom control, were obtained during the baseline visit. According to Global Initiative for Asthma criteria,^1^ asthma symptom control was classified as well controlled, partly controlled, and uncontrolled based on the assessment of the past 4 weeks: daytime asthma symptoms, any night waking due to asthma, short-acting β2-agonist reliever for symptoms, and any activity limitation due to asthma. The study was approved by the institutional review board of Peking University Health Science Center (approval No. IRB00001052-14090; dated March 11, 2015), and all participants provided written informed consent. The study was performed in strict accordance with the *Declaration of Helsinki* formulated by the World Medical Association and follows the Strengthening the Reporting of Observational Studies in Epidemiology (STROBE) Statement (**Additional file 1**).^24^

Thirty-two patients with asthma were recruited from Peking University Third Hospital from November 2015 to November 2016. Lung function data and ACT scores were repeatedly collected 2–13 times every month for each patient (totaling 266 visits).

### ACT evaluation

The ACT consists of five questions. The first four questions assess asthma symptoms over the past 4 weeks, including limitations in daily activities, dyspnea, nocturnal symptoms, and utilization of rescue medications. The fifth question reflects the patient’s self-assessment of overall asthma control level during the same period.^15^ Each item is scored from 1 to 5, with a total score ranging from 5 to 25. Higher scores indicate better asthma control.^25^

### Lung function measurements

Lung function was measured according to the 2019 guideline of the American Thoracic Society and European Respiratory Society.^26^ Data on lung function were obtained using a calibrated computerized spirometer (Elite Series Plethysmograph, MEDGRAPHICS Ltd., Saint Paul, MN, USA). The recorded parameters included FEV1, percentage of predicted FEV1 (FEV1%), forced vital capacity (FVC), FEV1/FVC, forced expiratory flow at 25% of the FVC (FEF 25), forced expiratory flow at 50% of the FVC (FEF 50), and mean forced expiratory flow between 25% and 75% of the FVC (FEF 25–75). These parameters were measured again approximately 20 ± 5 minutes after inhalation of 200 μg albuterol (Glaxo Wellcome, S.A., Aranda de Duero Burgos, Spain).

### Air pollutant measurements

Daily average concentrations of PM_2.5_, PM_10_, SO_2_, NO_2_, and CO were collected from November 2015 to December 2016 using data provided by the Beijing Municipal Ecological and Environmental Monitoring Center (http://zx.bjmemc.com.cn/). For each participant, pollutant data were obtained from the center air-monitoring station closest to the home address. Moving average air pollutant concentrations were calculated using daily data from days 1 to 7 before each clinical visit day for everyone.

### Statistical analysis

The linear mixed-effect models in R version 3.3.2 (https://www.r-project.org/) were used to assess the relationship between air pollutants and clinical effect indicators, including ACT scores and lung function.^27^,^28^,^29^ Because the ACT scores and lung function were log-normally distributed, the logarithms of the variables were used. All models included a random intercept for each study participant. The health indices were regressed on the moving average concentrations of exposures from days 1 to 7 prior to each measurement to investigate the potential cumulative exposure effects. The results were expressed as estimated percent changes in the ACT scores and lung function per interquartile range (IQR) increase in air pollutant concentrations. The estimated percent changes were calculated as [10^(β × IQR)^ – 1] × 100%, with 95% confidence intervals (CIs) calculated as {10^[IQR × (β ± 1.96 × SE)]^ – 1} × 100%, where β is the effect estimate and SE is the standard error.

Multivariable-adjusted models were used to control for potential confounding variables. Individual characteristics of the participants, including age, sex, and body mass index, were adjusted. A day-of-study variable, squared day-of-study variable, and day-of-week variable were included as fixed-effect terms to control for long-term time trends and weekly temporal variation. To adjust for disease status, asthma severity stage was included in the models. Both single- and two-pollutant models were used to explore the consistency of the exposure effects. Spearman correlation analysis was used to analyze the correlation between two pollutants. A *P* value < 0.05 was considered statistically significant.

## Results

### Participant characteristics and air pollutant levels

**Table 1** presents the baseline characteristics of the 32 patients with asthma (17 males and 15 females), with a mean age of 45.0 ± 11.7 years. Seven patients were assessed 2–5 times, 12 were assessed 6–9 times, and 13 were assessed 10–13 times, totaling 266 visits. Among the patients, 75% reported no history of smoking, 50% reported a history of allergy, and 69% experienced allergic rhinitis. The median duration of asthma was 1.5 years, with 62% of patients having partly asthma symptom controlled and 31% uncontrolled. The mean ACT score, FEV1%, FEV1/FVC, and fractional exhaled nitric oxide were 18.1 ± 4.5, 73.3 ± 23.6%, 67.4 ± 13.7%, and 50.6 ± 35.6 ppb, respectively. We collected lung function data 2–8 times per patient. **Table 2** presents these lung function measurements.

**Table 1:** Baseline characteristics of the participants.

Variable	Value
Sex (male/female)	17/15
Age (yr)	45.0±11.7
BMI (kg/m^2^)	25.5±3.9
Smoking status	
Current smoker	3 (9)
Ex-smoker	5 (16)
Never smoke	24 (75)
Passive smoking	8 (25)
History of allergy	16 (50)
Family history	
Chronic bronchitis	4 (13)
COPD	1 (3)
Asthma	1 (3)
Lung cancer	1 (3)
Comorbidities	
Allergic rhinitis	22 (69)
Hyperlipidemia	5 (16)
Hypertension	4 (13)
Hyperuricemia	2 (6)
Peptic ulcer	2 (6)
Angina	1 (3)
Hypothyroidism	1 (3)
Age of diagnose (yr)	39.6±12.2
Duration of disease (yr)	1.5 (0, 8)
Acute exacerbation in the previous year	
0	27 (84)
1	3 (9)
2	2 (6)
Severity	
Intermittent condition	3 (9)
Mild persistent	12 (38)
Moderate persistent	4 (13)
Severe persistent	13 (41)
Asthma Symptom Control	
Well controlled	2 (6)
Partly controlled	20 (63)
Uncontrolled	10 (31)
ACT score	18.1±4.5
FEV1%	73.3±23.6
FEV1/FVC	67.4±13.7
FeNO (ppb)	50.6±35.6

Data are expressed as number (sex), mean ± SD (age, BMI, age of diagnose, ACT score, FEV1%, FEV1/FVC, and FeNO), number (percentage) (smoking status, passive smoking, history of allergy, family history, comorbidities, acute exacerbation in the previous year, severity, and Asthma Symptom Control), or Median (quartiles) (duration of disease). ACT: Asthma Control Test; BMI: body mass index; COPD: chronic obstructive pulmonary disease; FeNO: fractional exhaled nitric oxide; FEV1: forced expiratory volume in 1 second; FVC: forced vital capacity.

**Table 2:** Lung function (pre-bronchodilator) of the participants

	Mean ± SD	Median	25^th^–75^th^
FEV1 (L)	2.5±0.9	2.4	2.0–3.0
FEV1%	75.6±25.1	78.5	58.6–92.0
FVC (L)	3.6±1.1	3.4	2.8–4.1
FVC%	91.8±20.8	93.0	78.0–103.0
FEV1/FVC (%)	67.9±14.7	70.0	57.0–79.0
FEF 25 (L/s)	4.5±2.5	4.3	2.2–6.6
FEF 25 (%)	69.5±37.3	68.0	37.1–101.5
FEF 50 (L/s)	2.4±1.5	2.1	1.3–3.5
FEF 50 (%)	51.8±31.2	47.0	26.0–73.6
FEF 25-75 (L/s)	1.9±1.1	1.6	1.1–2.8
FEF 25-75 (%)	58.2±34.9	50.0	30.5–82.0

FEF: Forced expiratory flow; FEV1: forced expiratory volume in 1 second; FVC: forced vital capacity.

**Table 3** presents the distribution of air pollutant concentrations on visit day. Spearman correlation analysis was performed to assess the relationship between each two air pollutants on visit day (**Table 4**). Strong positive associations were observed among the pollutants (*r* = 0.48–0.89, *P* < 0.01). PM_2.5_ presented the strongest correlation with PM_10_ (*r* = 0.89), followed by CO (*r* = 0.81). **Table 5** presents 1-day to 7-day moving average concentrations of pollutants prior to visit.

**Table 3:** Distribution of air pollutant levels on visit day

Pollutants	Mean±SD	Percentiles					IQR
		Minimum	25^th^	50^th^	75^th^	Maximum	
PM_2.5_ (μg/m^3^)	71.8±73.4	6.0	22.5	46.0	97.0	570	74.5
PM_10_ (μg/m^3^)	88.4±65.6	7.0	42.0	72.0	120.0	410.0	78.0
SO_2_ (μg/m^3^)	11.1±11.9	2.0	3.0	7.0	13.0	64.0	10.0
NO_2_ (μg/m^3^)	60.3±30.3	11.0	39.0	55.0	76.0	183.0	37.0
CO (mg/m^3^)	1.3±1.2	0.2	0.6	0.9	1.3	8.9	0.7

CO: Carbon monoxide; IQR: interquartile range; NO_2_: nitrogen dioxide; PM_10_: particulate matter with aerodynamic diameter less than or equal to 10 μm; PM_2.5_: particulate matter with aerodynamic diameter less than or equal to 2.5 μm; SO_2_: sulfur dioxide.

**Table 4:** Spearman correlations between air pollutant levels on visit day

Pollutants	PM_2.5_ (μg/m^3^)	PM_10_ (μg/m^3^)	SO_2_ (μg/m^3^)	NO_2_ (μg/m^3^)	CO (mg/m^3^)
PM_2.5_ (μg/m^3^)	1.0				
PM_10_ (μg/m^3^)	0.89**	1.0			
SO_2_ (μg/m^3^)	0.48**	0.47**	1.00		
NO_2_ (μg/m^3^)	0.72**	0.71**	0.60**	1.0	
CO (mg/m^3^)	0.81**	0.66**	0.60**	0.71**	1.0

**P < 0.01. CO: Carbon monoxide; NO_2_: nitrogen dioxide; PM_10_: particulate matter with aerodynamic diameter less than or equal to 10 μm; PM_2.5_: particulate matter with aerodynamic diameter less than or equal to 2.5 μm; SO_2_: sulfur dioxide.

**Table 5:** 1-day to 7-day moving averages of air pollutants prior to visit

Average	1 d	2 d	3 d	4 d	5 d	6 d	7 d
PM_2.5_ (μg/m^3^)	73.4±65.0	73.7±62.9	73.7±57.9	76.6±53.0	79.4±50.6	80.4±50.4	79.5±48.0
PM_10_ (μg/m^3^)	95.3±66.9	98.4±73.6	97.1±68.8	99.7±65.9	100.0±61.6	104.7±82.1	102.9±80.2
SO_2_ (μg/m^3^)	11.2±10.8	11.2±10.1	11.2±9.5	11.3±9.3	11.5±9.1	11.4±8.8	11.3±8.4
NO_2_ (μg/m^3^)	59.6±28.3	59.2±26.7	59.2±24.6	59.6±23.3	60.6±22.8	60.8±22.7	60.4±21.9
CO (mg/m^3^)	1.32±1.21	1.31±1.15	1.31±1.05	1.33±0.99	1.36±0.96	1.38±0.97	1.37±0.95

CO: Carbon monoxide; NO_2_: nitrogen dioxide; PM_10_: particulate matter with aerodynamic diameter less than or equal to 10 μm; PM_2.5_: particulate matter with aerodynamic diameter less than or equal to 2.5 μm; SO_2_: sulfur dioxide.

### Associations between air pollutant concentrations and the ACT score

**Figure 2:**
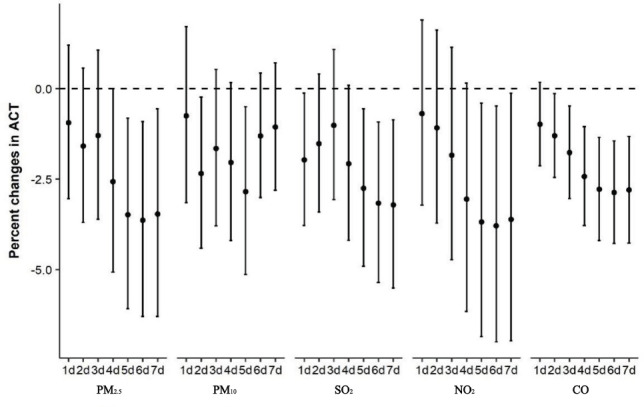
Estimated percent changes with 95% CIs in the ACT scores associated with IQR increases in air pollutant concentrations from 1-day to 7-day moving averages prior to the assessment of ACT scores in the single-pollutant models. ACT: Asthma Control Test; CI: confidence interval; CO: carbon monoxide; IQR: interquartile range; NO_2_: nitrogen dioxide; PM_10_: particulate matter with aerodynamic diameter less than or equal to 10 μm; PM_2.5_: particulate matter with aerodynamic diameter less than or equal to 2.5 μm; SO_2_: sulfur dioxide.

**Figure 2** presents the estimated percent changes in ACT scores associated with IQR increases in air pollutant concentrations in the single-pollutant models. Elevated concentrations of PM_2.5_, PM_10_, SO_2_, NO_2_, and CO were associated with reductions in ACT scores. The greatest decreases in ACT scores occurred at the 6-day moving average for PM_2.5_, NO_2_, and CO, 5-day moving average for PM_10_, and 7-day moving average for SO_2_. Specifically, per IQR increases in PM_2.5_ (74.5 μg/m^3^, 6-day), PM_10_ (78 μg/m^3^, 5-day), SO_2_ (10 μg/m^3^, 7-day), NO_2_ (37 μg/m^3^, 6-day), and CO (0.7 mg/m^3^, 6-day) were significantly associated with ACT scores reductions of 3.6% (95% CI: –6.3%, –0.9%), 2.8% (95% CI: –5.1%, –0.5%), 3.2% (95% CI: –5.5%, –0.9%), 3.8% (95% CI: –7.0%, –0.5%), and 2.9% (95% CI: –4.3%, –1.4%), respectively.

**Figure 3:**
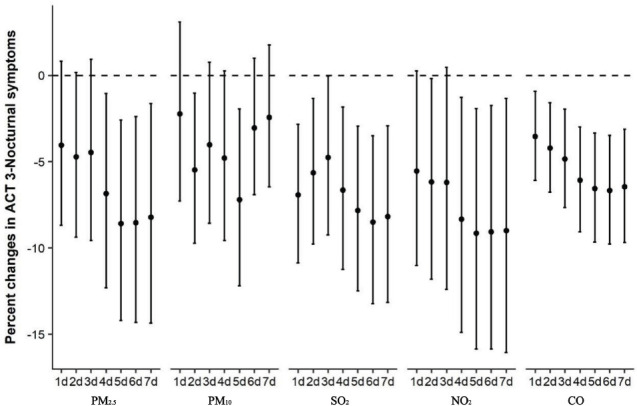
Estimated percent changes with 95% CIs in the ACT scores for nocturnal symptoms associated with IQR increases in air pollutant concentrations from 1-day to 7-day moving averages prior to the assessment of ACT in the single-pollutant models. ACT: Asthma Control Test; CI: confidence interval; CO: carbon monoxide; IQR: interquartile range; NO_2_: nitrogen dioxide; PM_10_: particulate matter with aerodynamic diameter less than or equal to 10 μm; PM_2.5_: particulate matter with aerodynamic diameter less than or equal to 2.5 μm; SO_2_: sulfur dioxide.

**Figure 4:**
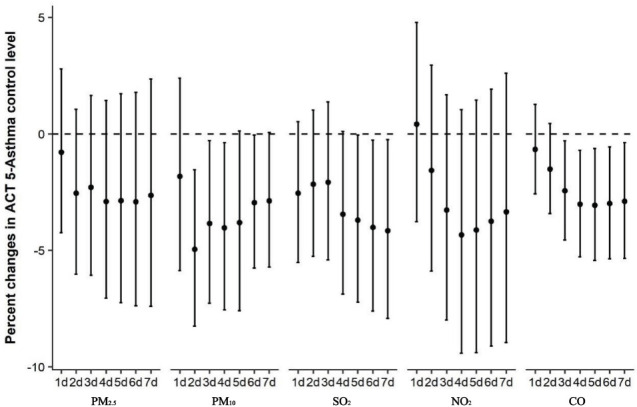
Estimated percent changes with 95% CIs in the ACT scores for asthma control level associated with IQR increases in air pollutant concentrations from 1-day to 7-day moving averages prior to the assessment of ACT scores in the single-pollutant models. ACT: Asthma Control Test; CI: confidence interval; CO: carbon monoxide; IQR: interquartile range; PM_10_: particulate matter with aerodynamic diameter less than or equal to 10 μm; PM_2.5_: particulate matter with aerodynamic diameter less than or equal to 2.5 μm; NO_2_: nitrogen dioxide; SO_2_: sulfur dioxide.

In the five ACT questions, higher concentrations of PM_2.5_, PM_10_, SO_2_, NO_2_, and CO were associated with lower ACT scores for nocturnal symptoms (the third question), as presented in **Figure 3**. Moreover, higher concentrations of PM_10_, SO_2_, and CO were associated with lower ACT scores for asthma control level (the fifth question), as presented in **Figure 4**.

**Figure 5:**
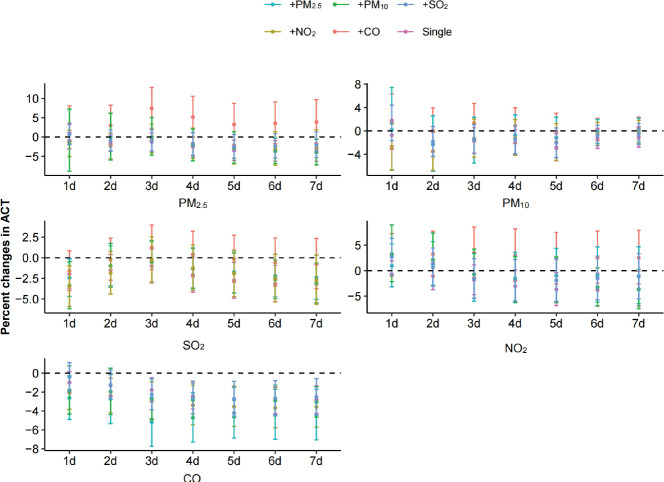
Estimated percent changes with 95% CIs in the ACT associated with increased IQR in air pollutant concentrations from 1-day to 7-day moving averages in the single- and two-pollutant models. ACT: Asthma Control Test; CI: confidence interval; CO: carbon monoxide; IQR: interquartile range; NO_2_: nitrogen dioxide; PM_10_: particulate matter with aerodynamic diameter less than or equal to 10 μm; PM_2.5_: particulate matter with aerodynamic diameter less than or equal to 2.5 μm; SO_2_: sulfur dioxide.

Estimated percent changes in the ACT associated with IQR increases in air pollutant concentrations in the single- and two-pollutant models are presented in **Figure 5** and **Additional Table 1**. In the two-pollutant models, the estimated changes in ACT scores associated with PM_2.5_, controlling for PM_10_, were generally greater than those correlated to PM_2.5_ in the single-pollutant models. Similarly, the estimated changes in ACT score associated with PM_10_, when controlling for NO_2_, were greater than those correlated to PM_10_ in the single-pollutant models. After adjusting for PM_10_ or NO_2_, the estimated changes in ACT scores associated with SO_2_ increased. In both single- and two-pollutant models, higher CO levels were associated with lower ACT scores. Moreover, after controlling for PM_2.5_, PM_10_, or NO_2_, the estimated changes in ACT scores associated with CO increased. In contrast, in other two-pollutant models, the estimated changes in ACT scores were generally weaker than those observed in the single-pollutant models.

**Additional Table 1:** Estimated percent changes with 95% CIs in the ACT associated with increased IQR in air pollutant concentrations from 1-day to 7-day moving averages in the single- and two-pollutant models

Pollutants	1-d	2-d	3-d	4-d	5-d	6-d	7-d
PM_2.5_							
Single	−0.9 (−3.0, 1.2)	−1.6 (−3.7, 0.6)	−1.3 (−3.6, 1.1)	−2.6 (−5.1, −0.0)	−3.5 (−6.1, −0.8)	−3.6 (−6.3, −0.9)	−3.5 (−6.3, −0.6)
+PM_10_	−1.1 (−8.9, 7.3)	−0.1 (−6.0, 6.2)	0.04 (−4.8, 5.1)	−2.1 (−6.2, 2.1)	−3.0 (−6.8, 1.4)	−3.7 (−6.9, −0.3)	−4.0 (−7.2, −0.6)
+SO_2_	0.7 (−2.0, 3.4)	−0.9 (−3.6, 1.8)	−1.0 (−3.7, 1.8)	−1.8 (−4.7, 1.2)	−2.5 (−5.5, 0.7)	−2.3 (−5.4, 0.9)	−2.0 (−5.3, 1.3)
+NO_2_	−1.7 (−5.1, 1.8)	−2.4 (−5.7, 1.1)	−0.4 (−4.1, 3.4)	−1.7 (−5.5, 2.3)	−2.9 (−7.0, 1.4)	−3.1 (−7.4, 1.4)	−2.8 (−7.3, 1.9)
+CO	3.4 (−1.0, 8.1)	3.0 (−2.0, 8.3)	7.4 (2.3, 12.9)	5.2 (0.01, 10.6)	3.3 (−1.9, 8.7)	3.6 (−1.7, 9.1)	3.9 (−1.6, 9.7)
PM_10_							
Single	−0.8 (−3.2, 1.7)	−2.3 (−4.4, −0.2)	−1.7 (−3.8, 0.5)	−2.0 (−4.2, 0.2)	−2.8 (−5.1, −0.5)	−1.3 (−3.0, 0.4)	−1.1 (−2.8, 0.7)
+PM_2.5_	0.2 (−6.6, 7.4)	−2.3 (−6.9, 2.6)	−1.7 (−5.5, 2.3)	−0.7 (−4.1, 2.7)	−1.2 (−4.6, 2.3)	−0.1 (−2.1, 1.9)	0.1 (−1.9, 2.2)
+SO_2_	1.3 (−1.7, 4.4)	−1.8 (−4.3, 0.7)	−1.4 (−3.9, 1.1)	−1.5 (−3.9, 0.9)	−2.1 (−4.6, 0.5)	−0.8 (−2.6, 1.0)	−0.5 (−2.3, 1.3)
+NO_2_	−2.8 (−6.8, 1.3)	−3.5 (−6.8, −0.0)	−1.3 (−4.5, 2.0)	−1.2 (−4.2, 1.9)	−2.0 (−5.1, 1.3)	−0.5 (−2.4, 1.5)	−0.2 (−2.2, 1.8)
+CO	−2.5 (1.8, 6.3)	−3.6 (0.1, 4.0)	−1.9 (1.4, 4.7)	−2.1 (0.9, 4.0)	−3.1 (0.1, 3.0)	−1.7 (0.2, 2.2)	−1.5 (0.4, 2.4)
SO_2_							
Single	−2.0 (−3.8, −0.1)	−1.5 (−3.4, 0.4)	−1.0 (−3.1, 1.1)	−2.1 (−4.2, 0.1)	2.8 (−4.9, 0.6)	−3.2 (−5.4, −0.9)	−3.2 (−5.5, 0.9)
+PM_2.5_	−2.4 (−4.7, −0.1)	−1.0 (−3.4, 1.4)	−0.5 (−3.0, 2.0)	−1.3 (−3.7, 1.2)	−1.7 (−4.2, 0.9)	−2.1 (−4.8, 0.4)	−2.4 (−5.0, 0.3)
+PM_10_	−3.4 (−6.2, 0.5)	−1.0 (−3.6, 1.8)	−0.5 (−3.0, 2.1)	−1.3 (−3.7, 1.1)	−1.9 (−4.3, 0.6)	−2.6 (−5.0, −0.3)	−3.1 (−5.5, −0.6)
+NO_2_	−3.5 (−5.9, −1.0)	−1.9 (−4.4, 0.8)	−0.2 (−3.0, 2.6)	−1.2 (−4.0, 1.5)	−1.9 (−4.7, 0.9)	−2.5 (−5.4, 0.4)	−2.7 (−5.6, 0.3)
+CO	−1.6 (−4.0, 0.9)	−0.2 (−2.7, 2.4)	1.3 (−1.4, 4.0)	0.4 (−2.3, 3.2)	−0.2 (−3.0, 2.7)	−0.6 (−3.4, 2.4)	−0.7 (−3.8, 2.4)
NO_2_							
Single	−0.7 (−3.2, 1.9)	−1.1 (−3.7, 1.6)	−1.8 (−4.7, 1.1)	−3.1 (−6.2, 0.2)	−3.7 (−6.8, −0.4)	−3.8 (−7.0, −0.5)	−3.6 (−7.0, −0.1)
+PM_2.5_	1.0 (−3.2, 5.3)	1.3 (−3.0, 5.7)	−1.4 (−6.0, 3.4)	−1.5 (−6.3, 3.6)	−1.0 (−6.1, 4.4)	−1.0 (−6.2, 4.7)	−1.0 (−6.5, 4.7)
+PM_10_	3.3 (−2.1, 9.0)	2.1 (−2.9, 7.4)	−0.7 (−5.4, 4.2)	−1.8 (−6.2, 2.8)	−1.9 (−6.3, 2.7)	−3.3 (−6.9, 0.6)	−3.7 (−7.5, 0.3)
+SO_2_	2.7 (−0.8, 6.4)	0.7 (−2.9, 4.5)	−1.6 (−5.4, 2.4)	−1.9 (−5.9, 2.2)	−1.9 (−6.0, 2.4)	−1.5 (−5.7, 2.9)	−1.2 (−5.5, 3.4)
+CO	3.0 (−1.1, 7.2)	3.2 (−1.1, 7.8)	3.5 (−1.3, 8.6)	3.0 (−2.0, 8.2)	2.3 (−2.6, 7.5)	2.5 (−2.5, 7.8)	2.5 (−2.6, 7.9)
CO							
Single	−1.0 (−2.1, 0.2)	−1.3 (−2.5, −0.1)	−1.8 (−3.0, −0.5)	−2.4 (−3.8, −1.0)	−2.8 (−4.2, −1.3)	−2.9 (−4.3, −1.4)	−2.8 (−4.3, −1.3)
+PM_2.5_	−2.6 (−4.9, −0.3)	−2.7 (−5.3, −0.0)	−5.2 (−7.7, −2.5)	−4.7 (−7.3, −2.1)	−4.2 (−6.9, −1.5)	−4.4 (−7.0, −1.7)	−4.4 (−7.1, −1.7)
+PM_10_	−1.8 (−4.3, 0.8)	−2.0 (−4.4, 0.5)	−2.7 (−4.8, −0.6)	−2.8 (−4.7, −0.9)	−2.8 (−4.7, −0.9)	−2.9 (−4.5, −1.3)	−3.1 (−4.6, −1.4)
+SO_2_	−0.4 (−1.9, 1.1)	−1.2 (−2.7, 0.3)	−2.2 (−3.9, −0.6)	−2.6 (−4.3, −0.8)	−2.7 (−4.5, −0.9)	−2.6 (−4.5, −0.8)	−2.5 (−4.4, −0.6)
+NO_2_	−2.1 (−3.8, −0.3)	−2.4 (−4.2, −0.5)	−2.9 (−5.0, −0.9)	−3.4 (−5.5, −1.3)	−3.5 (−5.6, −1.4)	−3.7 (−5.8, −1.5)	−3.6 (−5.7, −1.4)

ACT: Asthma Control Test; CI: confidence interval; CO: carbon monoxide; IQR: interquartile range; PM_10_: particulate matter with aerodynamic diameter less than or equal to 10 μm; PM_2.5_: particulate matter with aerodynamic diameter less than or equal to 2.5 μm; NO_2_: nitrogen dioxide; SO_2_: sulfur dioxide.

### Associations between air pollutant concentrations and lung function

**Figure 6:**
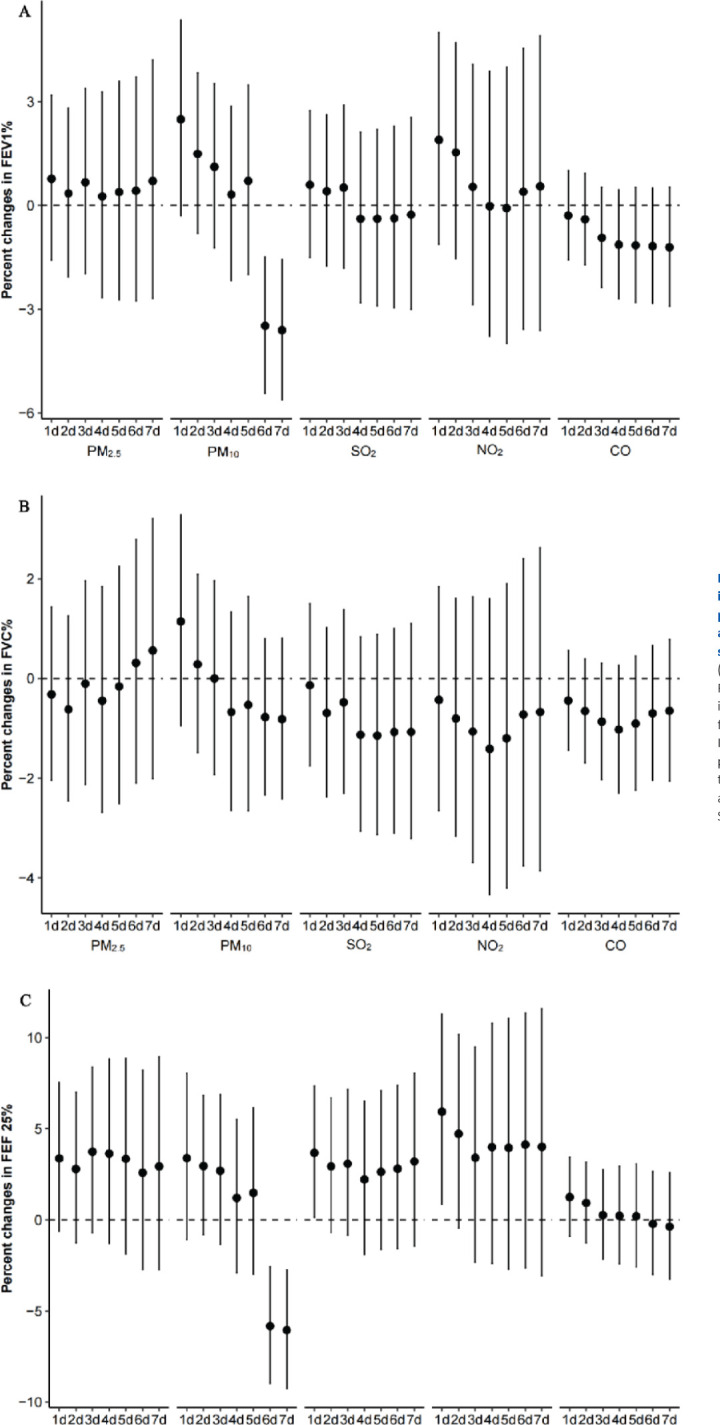
Estimated percent changes with 95% CIs in lung function associated with IQR increases in air pollutant concentrations from 1-day to 7-day moving averages prior to lung function assessment in the single-pollutant models. (A) Percent changes in FEV1%. (B) Percent changes in FVC%. (C) Percent changes in FEF 25%. CI: Confidence interval; CO: carbon monoxide; FEF: Forced expiratory flow; FEV1: forced expiratory volume in 1 second; IQR: interquartile range; NO_2_: nitrogen dioxide; PM_10_: particulate matter with aerodynamic diameter less than or equal to 10 μm; PM_2.5_: particulate matter with aerodynamic diameter less than or equal to 2.5 μm; SO_2_: sulfur dioxide.

Elevated PM_10_ concentrations were associated with reduced lung function in the single-pollutant models (**Figure 6**). The greatest reductions in FEV1% and FEF 25% were observed for the 7-day moving average of PM_10_. Specifically, an IQR increase of 78 μg/m^3^ in the 7-day moving average PM_10_ concentration was significantly associated with reductions of 3.6% (95% CI: –5.6%, –1.5%) in FEV1% and 6.0% (95% CI: –9.2%, –2.7%) in FEF 25%. No significant relationships were found between other pollutants and lung function.

## Discussion

Our study assessed the short-term effects of exposure to major air pollutants on respiratory symptoms and lung function among patients with asthma in Beijing. Increased concentrations of PM_2.5_, PM_10_, SO_2_, NO_2_, and CO were associated with worsened respiratory symptoms, as indicated by decreased ACT scores, particularly due to more frequent nocturnal symptoms and poorer asthma control levels. These associations remained significant in some two-pollutant models. Elevated PM_10_ concentrations were also associated with decreased lung function, including reductions in FEV1% and FEF 25%.

Although some studies reported no association between air pollution and respiratory symptoms,^30^,^31^ most studies supported a link between short-term exposure to air pollutants and worsening asthma symptoms in patients with asthma. For instance, the odds ratios of the combined outcome for wheezing and/or difficulty in breathing per 10 µg/m³ increase in mean 24-hour pollutant exposure were 1.07 for PM_10_, 1.30 for NO_2_, and 1.37 for SO_2_. This result indicated a moderately strong association between these pollutants and dyspnea in children and adolescents with asthma.^32^ Additionally, outdoor concentrations of PM_2.5_, PM_10_, and ozone were linked to an increased risk of respiratory symptoms.^33^ In another study, exposure to PM_10_ was associated with cough and phlegm. In the most polluted areas, exposure to PM_10_ was correlated to wheezing.^34^ Our findings are consistent with those of previous studies. We found that increased air pollution was associated with decreased ACT scores in adults with asthma. The Asthma Control Questionnaire is another tool used to evaluate asthma control. Zora et al.^35^ reported an association between the Asthma Control Questionnaire scores and air pollution levels among patients using inhaled corticosteroids. Our study further revealed that the reduction in ACT scores correlated with air pollutants was higher in longer than in shorter time intervals. The correlated decrease in ACT scores reached its maximum at a 5-day moving average for PM_10_, a 6-day moving average for PM_2.5_, NO_2_, and CO, and a 7-day moving average for SO_2_. These results suggest that exposure to ambient air pollution might have lagged effects on symptom burden. In our study, although ACT scores were used to evaluate symptoms in the preceding 4 weeks and the air pollutant exposures were measured only for the preceding 7 days, associations between them were still observed. He’s study^17^ and Maestrelli’s study^19^ showed similar results of associations of 24 hours personal PM_2.5_ exposure measured 1 and 4 days prior to the clinic visits or 24 hours PM_10_ personal exposure with ACT score. We guess the relationship between poorer ACT score and short-term increasing air pollutants exposure may be due to the participants living in a relatively fixed location and being exposed to a relatively stable and similar concentration of air pollutants for more than 1 week. However, this needs to be confirmed by long-term air pollutant exposure data. When applied appropriately, the ACT may serve as a practical tool to monitor symptom fluctuations caused by varying pollution levels in heavily polluted areas.

We found that PM_10_ had similarly negative effects on forced volumes, including FEV1%, which represented the function of the central airways and respiratory flow variables, and including FEF 25%, which represented the function of the peripheral airways. Moreover, the reduction in lung function correlated to PM_10_ was higher in longer than in shorter intervals. The associated reduction in FEV1% or FEF 25% reached its maximum at 7-day moving average. These results suggest that PM_10_ may have potential cumulative and lagging effects on lung function in individuals with asthma. There were no apparent associations between air pollutants except PM_10_ and lung function in our study. In contrast, a previous report indicated a significant negative association between the 5-day accumulated average of PM_2.5_, and FEV1 and FVC in children with asthma.^36^ Another study revealed that short-term exposure to PM_2.5_ may impair small airway function, reduce nasal microbiota evenness, and induce microbiota dysbiosis in children with asthma.^37^ There was also a strong association between the estimated NO_2_ exposure per 10 mg/m^3^ and lung function in school children, particularly with the following expiratory flows: –0.62% for FEV1/FVC, –62 mL/s for FEF25–75, and –85 mL/s for peak expiratory flow.^30^ Ierodiakonou et al.^20^ found that the same-day and 1-week average CO levels were inversely associated with post-bronchodilator FEV1% and FVC. In a meta-analysis, a 10 μg/m^3^ increase in NO_2_ level was associated with an 8 mL decrease in FEV1.^38^ These findings suggest that different pollutants may cause a reduction in lung function among patients with asthma. The mechanisms of air pollutants promoting the development and exacerbation of asthma include the induction of inflammation in airway epithelium, oxidative stress and DNA methylation.^39^ Air pollution promotes the release of epithelial cytokines, driving T helper 2 responses, and induces oxidative stress and the production of proinflammatory cytokines, which may be associated with the exacerbation of asthma.^40^ Duan’s study^41^ revealed a link been personal PM_2.5_ exposure and genome-wide DNA methylation in adult patients with asthma. In our study, the effects of different atmospheric pollutants on symptoms and lung function were inconsistent. The association between asthma control measures and lung function in cross-sectional analyses remains inconclusive.^42^ Some studies have reported weak to moderate correlations between ACT scores and FEV1, while others found no significant correlation between them.^42^

Our study has several strengths. First, we found that air pollution was negatively associated with lung function. Moreover, the air pollutant levels in Beijing were relatively high and exhibited significant variations, causing significant effects and facilitating their detection. However, our research has several limitations. First, the sample size was relatively small. Larger cohorts are needed to validate these results. Second, air pollution data were collected from the closest central air-monitoring stations, rather than from participant’s workplaces or personal monitoring devices, which may not reveal the exact relationship between pollutants and health outcomes. Third, our study did not account for factors such as temperature, humidity, and seasonal variation, all of which may influence the impact of air pollution on asthma symptoms and lung function. Fourth, the mechanisms by which air pollutants affect respiratory symptoms and lung function among patients with asthma was not assessed. Thus, further studies of this mechanism are warranted.

In conclusion, short-term exposure to high levels of ambient air pollutants, such as PM_2.5_, PM_10_, SO_2_, NO_2_, and CO, was associated with a significant reduction in ACT scores, and short-term exposure to PM_10_ might cause decreased lung function in adult patients with asthma. This study highlights the health risks posed by air pollution and underscores the need for strategies to protect the health of patients with asthma living in areas with high pollution levels.

## Additional files:

***Additional Table 1:*** *Estimated percent changes with 95% CIs in the ACT associated with increased IQR in air pollutant concentrations from 1-day to 7-day moving averages in the single- and two-pollutant models.*

***Additional file 1:*** *STROBE checklist.*

STROBE Statement—Checklist of items that should be included in reports of *cohort studies*

## Data Availability

*All relevant data are within the paper and its additional files.*
